# A Rare Case of Small-Cell Neuroendocrine Cancer of the Cervix: An Unexpected Diagnosis

**DOI:** 10.7759/cureus.34006

**Published:** 2023-01-20

**Authors:** Deepthy Balakrishnan, Saunri Hansadah, Pooja Sahu

**Affiliations:** 1 Department of Obstetrics and Gynecology, All India Institute of Medical Sciences, Bhubaneswar, Bhubaneswar, IND

**Keywords:** small cell neuroendocrine cervical cancer (scncc), postmenopausal bleeding, international federation of gynecology and obstetrics (figo), immunohistochemistry, cervical cancer

## Abstract

Cervical cancer is a significant healthcare problem worldwide, especially in developing countries. It is the second most common cause of cancer-related deaths in women. Small-cell neuroendocrine cancer of the cervix (SCNCC) accounts for about 1-3% of all cervical cancers. In this report, we present a case of a patient with SCNCC, whose disease had metastasized to the lungs even without an obvious growth in the cervix. A 54-year-old multiparous woman presented with post-menopausal bleeding for 10 days; she had a history of a similar episode in the past. Examination revealed an erythematous posterior cervix and upper vagina without any obvious growth. Histopathology showed SCNCC on the biopsy specimen. Following further investigations, the stage assigned was IVB, and she was started on chemotherapy. SCNCC is an extremely rare but highly aggressive type of cervical cancer and it requires a multidisciplinary therapeutic approach for an optimal standard of care.

## Introduction

Cervical cancer is a major healthcare concern globally, particularly in developing countries. It is the second most common cause of cancer-related deaths in women [1]. Small-cell neuroendocrine cancer of the cervix (SCNCC) accounts for about 1-3% of all cervical cancers [2]. As compared to other histological types of cervical cancers, SCNCC exhibits early distant metastasis and has a worse prognosis [3]. The reported five-year survival rate is nearly 36% in cases of SCNCC [2]. We present a patient with SCNCC, who had metastatic disease to the lungs even without any obviously visible growth in the cervix.

## Case presentation

**Figure 1:**
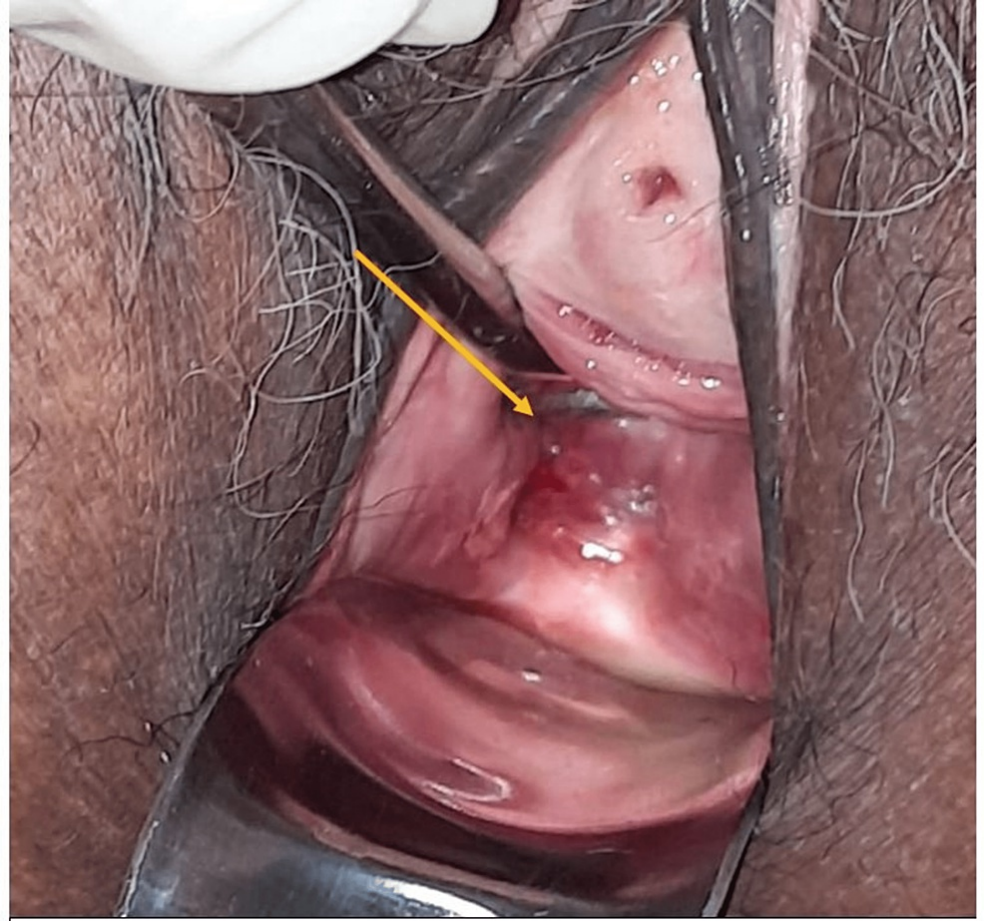
Erythematous cervix and posterior upper vagina on per speculum examination The yellow arrow indicates the erythematous posterior vaginal wall

A 54-year-old parous woman presented with post-menopausal bleeding for 10 days. She had experienced a similar episode a few years back. There was no history of postcoital bleeding, foul-smelling vaginal discharge, or any urinary or bowel complaints. Her previous menstrual cycles had been normal. She had never undergone screening for cervical cancer before. The general examination was unremarkable. On per speculum examination, the cervix was partially flushed with the vagina, and the posterior lip of the cervix and the adjacent upper vagina were erythematous and unhealthy looking (Figure 1). Per vaginal examination revealed an irregular, hard, and indurated area of about 4x3 cm involving the posterior cervix, posterior fornix, and posterior upper one-third of the vagina. The left parametrium appeared to be involved, and the right parametrium and rectal mucosa were free. A cervical punch biopsy was taken at the same sitting as her pap smear, which showed evidence of squamous cell carcinoma of the cervix.

**Figure 2:**
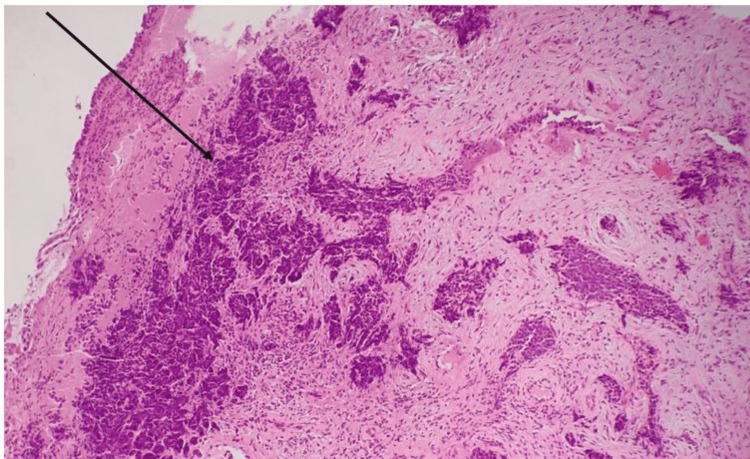
H&E stain 40x: tumor cells arranged in irregular islands The black arrow indicates tumor cells in irregular islands

**Figure 3:**
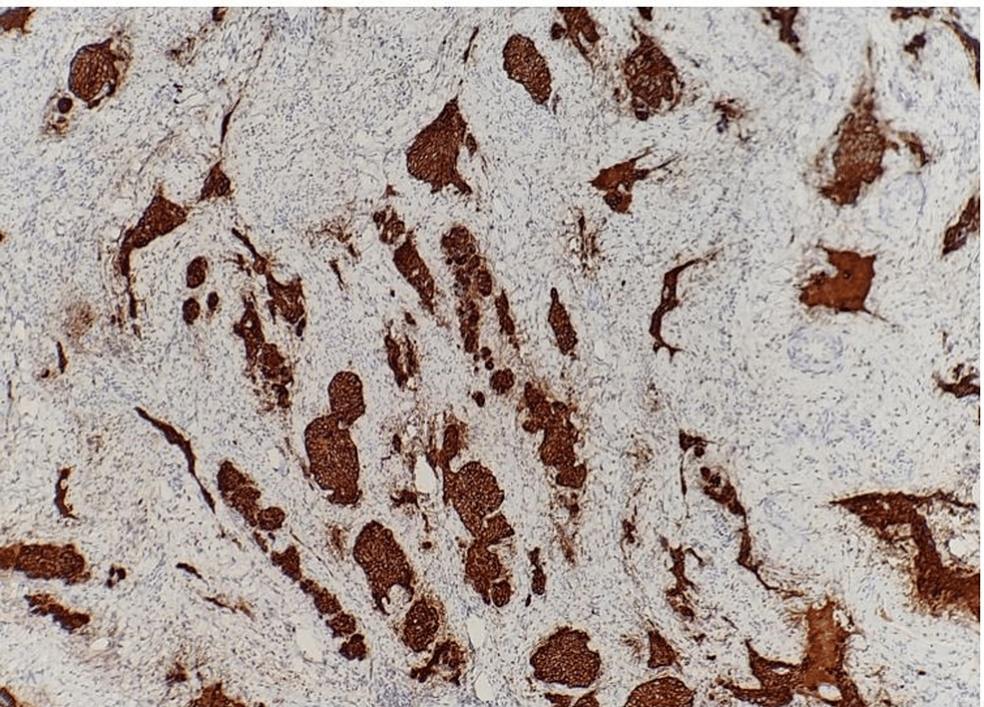
Immunostain 200x: tumor cells positive for pancytokeratin

Contrast-enhanced CT (CECT) of the abdomen and pelvis showed a heterogeneously enhancing lesion of 3.7x4.5x3.3 cm involving the cervix, lower uterine body, adjacent parametrium, and upper vagina. The involvement of the bilateral common iliac, obturator, and right parametrial and mesorectal nodes was also noted. A few suspicious deposits were seen in the lungs, which were subsequently confirmed to be metastatic deposits by high-resolution CT (HRCT) of the thorax. Histopathology confirmed the diagnosis of SCNCC, and immunohistochemistry was positive for cytokeratin, synaptophysin, and chromogranin but negative for P40 (Figures 2, 3). Based on the International Federation of Gynecology and Obstetrics (FIGO) classification, our patient's disease was classified as carcinoma cervix stage IVB, and she was started on a combination regimen of cisplatin and etoposide. She received three cycles of radiotherapy but was lost to follow-up after that.

## Discussion

Neuroendocrine tumors arise from the diffuse neuroendocrine cell system. The sites usually affected are the lungs and bronchi, small intestine, appendix, rectum, pancreas, and thymus, whereas the uterine cervix is an extremely uncommon site. SCNCCs are rare malignancies, accounting for 1-3% of all cervical tumors [2]. The prognosis of SCNCC is determined by the site of origin and histologic subtypes. Histopathologically, it has four subtypes: well-differentiated subtypes that include carcinoid and atypical carcinoid types, and poorly differentiated groups that include small-cell and large-cell tumors [2].

SCNCC displays characteristic clinical and biological properties of both cervical neoplasm and neuroendocrine small-cell cancer of other sites. It is locally aggressive and the human papillomavirus (HPV) is the causative organism as in cervical neoplasm, and it causes early metastasis and loss of heterozygosity at different loci as in neuroendocrine small cell cancer of other sites. It tends to have high mitotic activity, extensive tissue necrosis, lymphovascular space involvement (LVSI) at an early stage, and a strong HPV-18 association. In a case series by Giordano et al. [4], HPV DNA was observed in all three cases of neuroendocrine carcinomas by PCR analysis. However, in our case, HPV DNA analysis was not performed. The histologic differential diagnosis can include other small blue cell tumors arising from the cervix, such as lymphoma, basaloid squamous cell carcinoma, embryonal rhabdomyosarcoma, and undifferentiated carcinoma. They can be differentiated by immunohistochemical analysis [5]. Neuroendocrine tumors show synaptophysin, CD56, and chromogranin positivity whereas Ki 67/MIB-1 labeling indicates their malignant characteristics in immunohistochemistry [3]. In the present case, immunohistochemistry was positive for cytokeratin, synaptophysin, and chromogranin while negative for P40.

The median age at which the patients usually present is in the fifth decade of life (range: 21-87 years); patients usually present with features similar to other forms of cervical cancers, such as vaginal bleeding or discharge and cervical growth on examination. It can also present with cachexia, loss of weight or appetite, abdominal pain, and symptoms due to distant metastasis in an advanced stage. In rare cases, patients can have hypercalcemia, Cushing's syndrome, and syndrome of inappropriate ADH secretion if endocrinologically active [6]. In a case reported by Dalli et al. [7] and in two cases out of three reported by Giordano et al. [4], the presentation was post-menopausal unproved vaginal bleeding, which is similar to our case.

The staging of SCNCC is similar to that of traditional cervical cancer. MRI is the gold standard imaging modality for SCNCC. Chan et al., in 2003, proposed an algorithm for the management of SCNCC [8]. In 2011, the Society of Gynecologic Oncology modified the approach and stated that in early stages (FIGO I-IIA), tumors <4 cm are amenable to surgery followed by adjuvant chemotherapy with etoposide/platinum-based agents, and tumors >4 cm could be considered for a neoadjuvant approach. Combination chemotherapy and chemoradiation were recommended for late-stage disease and for women not fit for surgery [2]. In most of the reported cases, the diagnosis was made in the advanced stage, and hence the primary treatment modality was systematic chemotherapy, as was in our case.

SCNCC does not follow the locoregional pattern of spread. The three-year survival rate in grade 1-2 disease is about 80% while it is nearly 38% in grade 3-4 [8]. The extra pelvic recurrence rate is very high with lungs, bones, and supraclavicular lymph nodes being the most common sites. Distant-site recurrence is more frequently observed (28%) than local recurrence (13%) [2]. The stage at presentation, size of the tumor, the presence and number of lymph node metastasis, pure small cell histology, and history of smoking determine the chances for the recurrence of the tumor [8,9].

## Conclusions

SCNCC is an extremely rare and highly aggressive type of cervical cancer. It is associated with a fatal clinical course, which is markedly worse when compared to squamous cell carcinoma and adenocarcinoma of the cervix. It can metastasize to lymph nodes and distant sites even in the early stages of the disease. Due to its aggressive course and early metastasis, it becomes inoperable if the diagnosis is delayed. Hence, early identification and intervention with a multidisciplinary therapeutic approach are keys to an optimal standard of care.
